# Macrocognition: From Theory to Toolbox

**DOI:** 10.3389/fpsyg.2016.00054

**Published:** 2016-01-29

**Authors:** Gary Klein, Corinne Wright

**Affiliations:** ^1^MacroCognition LLCWashington, DC, USA; ^2^ShadowBox LLCDayton, OH, USA

**Keywords:** NDM, ShadowBox, cognitive, decision making, complexity

## Abstract

We trace several trajectories—the evolution of field-based decision making models in the mid-1980s to the formation of the Naturalistic Decision Making movement in 1989, then the further broadening of NDM into Macrocognition in 2003, and finally the transition from macrocognitive models into a set of methods and tools to boost cognitive performance.

## Stage 1: Naturalistic decision making

During the 1980s, several researchers (Rasmussen, 1985; Cohen, 1986; Beach and Mitchell, 1987; Klein, 1989; Noble, 1989) independently began investigating the nature of decision making in work settings as opposed to laboratory, controlled settings. The NDM movement was catalyzed by a program established by Judith Orasanu at the Army Research Institute for the Behavioral and Social Sciences. Orasanu's program funded several of the investigators and also brought them together at periodic program reviews. A critical mass formed, leading to a 1989 workshop to prepare a book describing the NDM perspective. Approximately 30 researchers were invited to the meeting, including representatives from the US Army, Navy, and Air Force. The Navy was particularly interested in the topic because the Vincennes shoot down had occurred just a year earlier—an advanced AEGIS cruiser had shot down an Iranian commercial airliner, mistaking it for an attacking F-14. The Navy was shortly to initiate its own program of naturalistic decision research, Tactical Decision Making Under Stress (TADMUS).

**Figure 1:**
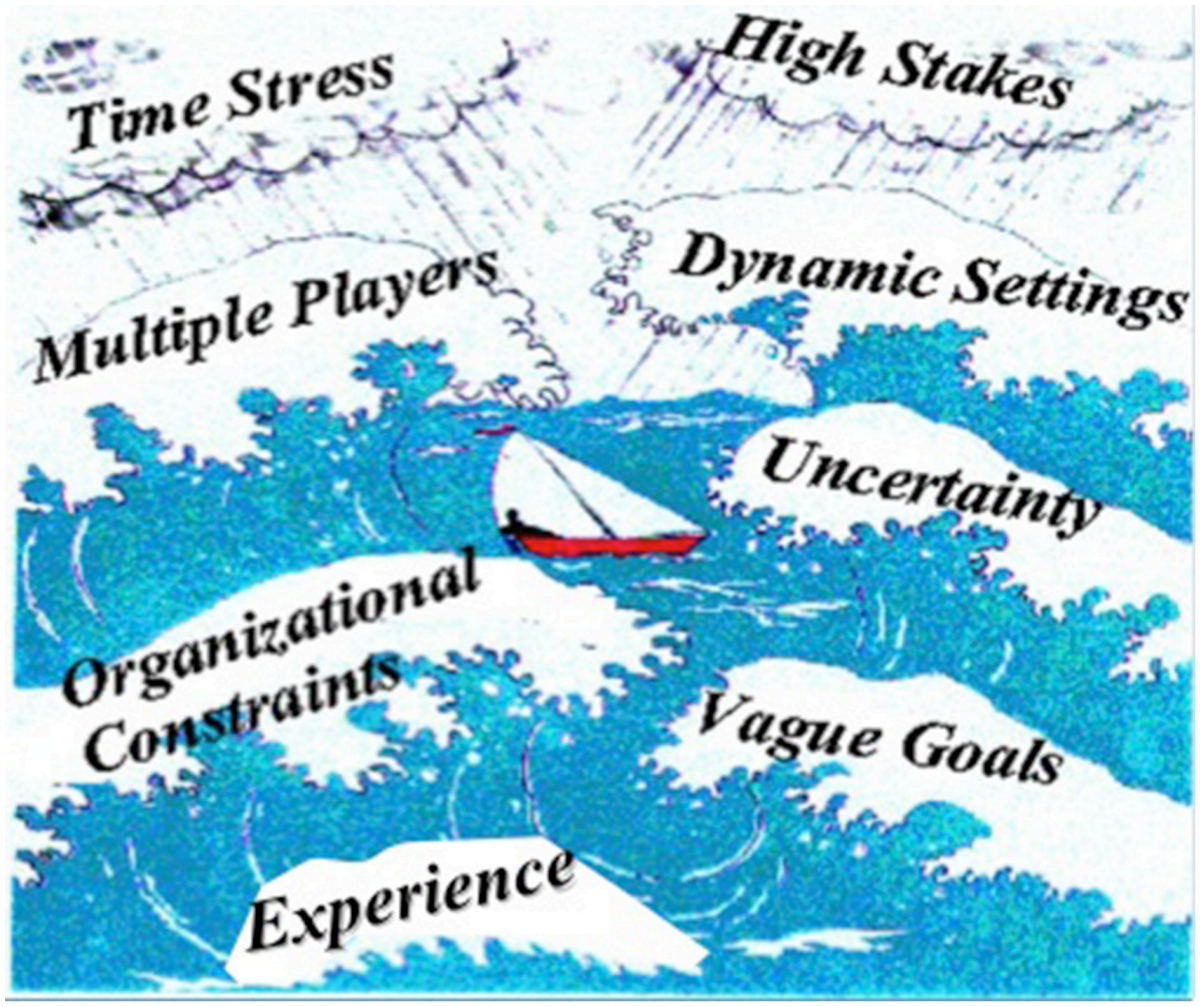
**NDM variables (Klein et al., 1993). Illustrated by David Sweeney**.

The 1989 workshop resulted in an edited book, *Decision making in action: Models and methods* (Klein et al., 1993). The central NDM theme was to stud decision making under complex conditions, with vague goals, organizational constraints, high stakes, and levels of experience not easily captured in controlled laboratory settings (see Figure 1).

The term “Naturalistic Decision Making” was coined at the 1989 workshop, mirroring a related emerging topic of interest in the psychology of learning, *Naturalistic Memory*, initiated by Ulric Neisser. Naturalistic Memory encompassed topics of everyday memory, autobiographical memory, and practical memory (Gruneberg et al., 1978; Neisser, 1982).

Yet Naturalistic Memory quickly faded, even though Neisser was an iconic figure whose 1967 book *Cognitive Psychology* had helped to establish a new discipline (Neisser, 1967). Why did this happen? One possibility is that because Neisser was so famous, his suggestion of studying memory under non-controlled conditions was seen as blasphemous. In 1989 the American Psychologist devoted a special issue to allow critics to explain why Neisser's project was misguided (e.g., Banaji and Crowder, 1989—“The bankruptcy of everyday memory”). Laboratory researchers ridiculed the notion that anything of use could be learned from studying memory in natural settings. It is jarring to read their comments, aimed at one of the giants of the field, insisting that the new methodology should not even be explored.

In contrast, NDM already had generated an important discovery. Klein et al. (1986) and Klein (1989) described how people were able to make decisions under time pressure and uncertainty— the Recognition-Primed Decision (RPD) model. This finding parried any criticism that there was nothing to be learned from studying decision making in a natural setting. The RPD model accounted for 80–90% of the decisions that firefighters made. And the RPD model could never have been discovered under laboratory conditions because the RPD model depended on experience that took 10–20 years to develop. Laboratory-based decision research gave college sophomores unfamiliar tasks in order to avoid any contaminating effects of prior experience that might add unwanted variability to the results. Findings supporting the RPD model were replicated by different research teams in different contexts: military leaders (Schmitt and Klein, 1999), firefighters, (Keren et al., 2013), and managers of offshore oil drilling platforms (Skriver, 1998). The RPD model itself was tested and received empirical support (Klein et al., 1995; Johnson and Raab, 2003).

Naturalistic Decision Making researchers study how people actually make decisions. The word “actually,” may discomfort laboratory researchers who can reasonably argue that even college students performing unfamiliar tasks are “actually” making decisions. However, many compromises have to be made to perform controlled experiments. The restriction on context, the absence of meaningful consequences, the use of tasks with well-defined goals, and particularly the elimination of expertise in studies presenting unfamiliar tasks, all raise doubts about whether the findings of these studies can be generalized to natural settings. Laboratory researchers can counter that the lack of controlled conditions also raise doubts about the results of NDM studies. We are not arguing that either tradition is the correct one. We merely assert that NDM projects offer unique opportunities for discoveries.

There was some criticism of NDM from laboratory-based decision researchers. Lipshitz et al. (2001) published a lead article in the *Journal of Behavioral Decision Making*, and an unprecedented number of researchers wrote commentaries, 16 in all, centered around the theme that NDM needed to mature as a discipline and establish more rigorous and controlled methods of investigation. The NDM community viewed these criticisms as misguided. If NDM researchers followed the critics' suggestions and performed studies under controlled conditions, they would no longer be doing NDM work. The scientific method begins with observing the phenomenon of interest, which is the core of NDM research.

Unlike Naturalistic Memory, which quickly faded, NDM thrived after the first NDM workshop in 1989. Thus, far, a total of 12 NDM conferences have been held. A Cognitive Engineering and Decision Making technical group, drawing on the NDM community, was established in 1995 within the Human Factors and Ergonomics Society. This group now has its own *Journal of Cognitive Engineering and Decision Making*.

## Stage 2: Macrocognitive models

**Figure 2:**
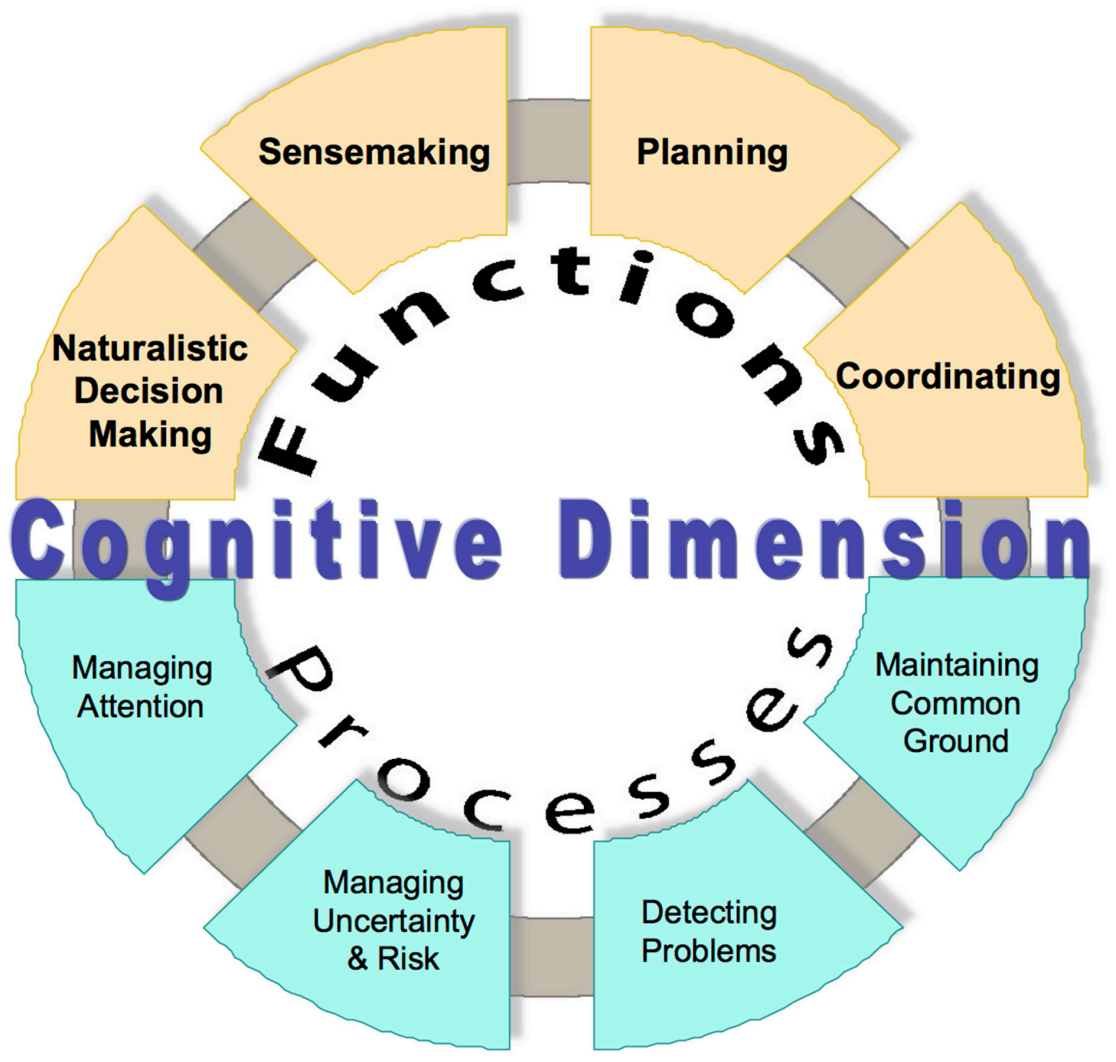
**Macrocognitive functions and processes**.

Because of the success of naturalistic inquiry into decision making, NDM researchers quickly began applying this approach to other cognitive phenomena, such as planning (e.g., Klein, 2007a,b), sensemaking (e.g., Klein et al., 2006), and uncertainty management (e.g., Lipshitz and Strauss, 1997), as well as the development of expertise itself (e.g., Klein, 1997). Figure 2 illustrates the range of cognitive functions and processes addressed by macrocognitive models (Klein et al., 2003).

Klein et al. (2000) differentiated macrocognition and microcognition. They defined macrocognition as the study of cognitive processes affecting people such as firefighters, pilots, nurses, and others who had to wrestle with difficult dilemmas in complex settings under time pressure and uncertainty. Microcognition was the study of the components of thinking such as working memory, and serial vs. parallel attentional processes. Other researchers had used the term macrocognition (e.g., Cacciabue and Hollnagel, 1995) in papers, but had not identified it as a separate field of study, an expansion of the NDM enterprise (for a fuller history, see Hoffman and McNeese, 2009).

The NDM community has now expanded its perspective beyond decision making, to cover the variety of macrocognitive models and to perform naturalistic studies of cognitive processes and variables. Sometimes, macrocognitive studies are performed under controlled conditions and the studies often involve the control and manipulation of variables. But the core of the work examines cognitive processes in complex contexts—in the context of the work environment. Macrocognitive studies usually address expertise, how it develops, what constitutes it, how it is used to perform challenging tasks. Sometimes researchers will investigate novices, to see how they differ from experts, how they approach tasks, and where they struggle.

The criticisms of NDM that have been raised by laboratory scientists also will apply to macrocognition. It is less concerned with testing hypotheses than with formulating useful models and theories. It is less concerned with precision than with plausibility. It is less concerned with normative or “rational” models than with descriptive models. It wallows in messy variables such as wicked problems with ill-defined goals, team and organizational constraints, uncertainty, and high stakes. It studies tasks for which there are no correct solutions, making it difficult to evaluate performance. Guilty as charged. These are the conditions in which we live and work, and macrocognitive research attempts to better understand them. Figure 1, a diagram originally formulated for NDM equally well illustrates the variables of interest for macrocognition.

Klein (2015) described the impact of the NDM/macrocognitive perspective, by cataloging the way this perspective has changed so many core beliefs previously held in the basic and applied communities.

We no longer claim that the only way to make a good decision is to generate several options and compare them to pick the best one (experienced decision-makers can draw on patterns to handle time pressure and never even compare options; Klein, 1989; Hoffman, 1992). We no longer believe that expertise is based on learning rules and procedures (it primarily depends on tacit knowledge, Klein and Hoffman, 1993). We no longer believe that projects must start with a clear description of the goal (many projects involve wicked problems and ill- defined goals, Hoffman, 2007). We no longer believe that people make sense of events by building up from data to information to knowledge to understanding (experienced personnel use their mental models to define what counts as data in the first place, Skriver et al., 2004; Schraagen et al., 2008). We no longer believe that insights arise by overcoming mental sets (they also arise by detecting contradictions and anomalies and by noticing connections, Klein, 2013). We no longer believe that we can reduce uncertainty by gathering more information (performance seems to go down when too much information is gathered—Uncertainty can stem from inadequate framing of data, not just from the absence of data, Cannon-Bowers et al., 1993; Omodei et al., 2005; Flin et al., 2008; Grossman et al., 2014). We no longer believe that we can improve performance by teaching critical thinking precepts such as listing assumptions (too often the flawed assumptions are ones we are not even aware of and would never list, Klein, 2011; Stanton et al., 2011; Hoffman et al., 2014).

Whereas the behavioral decision making community focuses on human limitations and seeks ways to reduce biases and mistakes, the NDM community, as it performs macrocognitive research, focuses on human capabilities and regards good performance as much more than the absence of mistakes. Good performance is also about discoveries and insights; it is about the strengths of decision makers, and the importance of experience. Experience serves a variety of functions including a larger repertoire of patterns and associated actions, a richer mental model of how things work to support inferential reasoning and sensemaking for diagnosis and anticipation.

## Stage 3: Macrocognitive methods and tools

Macrocognitive models lend themselves to a set of methods and tools that can be used in cognitively challenging activities. This third stage is about compiling a toolbox, not to do research, but to enhance performance.

A recent study illustrates how a macrocognitive perspective provides a unique diagnosis of a problem, not by blaming those committing the error (those on the sharp end, as James (Reason, 1990), would put it), but by investigating how conscientious employees could be making poor decisions (Multer et al., 2015; Safar et al., 2015). The problem was railroad crashes caused by locomotive engineers who failed to stop despite clear signals warning them of danger. It seemed obvious that the locomotive engineers were getting distracted or were failing to pay adequate attention. Strategies were devised to help the engineers, including a “Keep the Focus” program. However, Multer et al. (2015) viewed the inattention/distraction issue as a *symptom* of the problem, not as the *source* of the problem. They investigated the reasons for the inattentiveness, using interviews and field observations. They examined the sociotechnical context of the “Signals Passed at Danger” phenomenon. One finding was that each signal had several lights, including a red light, but the red light was always on! Thus, the red light provided no information. The other lights signaled whether to stop or proceed with caution.

Another finding was that central stations had outgrown their original design because railway traffic had increased. Space became more cramped, the switching arrangements had become more complex, and the viewing angles became more ambiguous as different signals were moved closer and closer, to the point that the engineers were not always sure which signal pertained to which line. Worse, because trains were now longer, signals were sometimes placed behind the train and out of sight of the engineers in the locomotive cab.

Holtrop et al. (2015) used Cognitive Task Analysis in the domain of healthcare. They were sending healthcare practitioners—nurses, aides, etc.—into the community to work with patients who had chronic illnesses such as cardiac disease and diabetes. But the effort was running into barriers because each clinic and practice had its own decision making style. So the project added a Cognitive Task Analysis training piece, a two-day workshop to train the outreach personnel in CTA methods in order to overcome the differences in decision making. The training was highly successful, and the recommendations gained greater acceptance. Accordinatly, the healthcare practitioners became advocates for front-end CTA analysis prior to initiating any new effort.

Many domains depend on training to get employees up to speed, but the training usually centers on rules and procedures. What is missing is a concern for the tough decisions employees will have to make once they complete their training: the difficult sensemaking they will face when confronted with ambiguous cues and erroneous data, the challenging problem detection when things are just starting to go wrong. The field of macrocognition is well suited for addressing cognitive training requirements.

Hoffman et al. (2014) provided an important resource—a compilation of best practices for accelerating the development of expertise. They identified strategies for practice and feedback, transfer, and retention, and also addressed team training issues. Expertise is central to all the macrocognitive processes. Tactics for speeding up expertise are essential macrocognitive tools.

The design of new systems, and the modification of existing systems, can benefit greatly from a macrocognitive perspective, and a variety of methods have emerged for injecting cognition into the design process (e.g., Militello and Hutton, 1998; Militello et al., 2010; Militello and Klein, 2013) and for making automation a team player (Klein et al., 2004). The intent here is to develop macrocognitive work systems.

One last example of a macrocognitive tool is the ShadowBox approach. ShadowBox® training is a scenario-based way to enable trainees to see the world through the eyes of experts without the experts having to be present. One of the bottlenecks of expert feedback is that there is a limited number of experts and their time is jealously guarded. ShadowBox presents challenging scenarios and intersperses decision points at which the participant is asked to rank a given set of options. These may be options about which course of action to choose, which goal to prioritize, which cues to monitor carefully, or which pieces of information to gather. A participant ranks the options and writes his/her rationale for the rankings. In preparation for the training session, a panel of subject-matter experts also has rank ordered the options and provided their rationale statements. At this point, the experts are no longer needed. The expert rankings are combined, and the rationale statements are synthesized. Once the participant provides rankings and rationale, he/she sees what the panel of experts ranked, and sees the experts' rationale, noticing what he/she had missed. In this way, the participant gets to see the scenario through the eyes of the experts, and gets a sense of the experts' mental models.

Hintze (2008), a Battalion Chief with the New York Fire Department originated the ShadowBox concept. Klein et al. (2013) elaborated on the ShadowBox concept. While it is primarily a means of providing cognitive training, it also can be used as a knowledge management tool to capture the wisdom of experts in the form of the rankings and reasons they provide. A third use of ShadowBox is for assessment, using a participant's rankings and rationale to evaluate competence. And a fourth use is for better teamwork. Here, team members identify how they would react at critical moments in a scenario, and they predict how their partners would react. Then each partner sees what the other would do, and what the other expected. In this way, teams can get better at predicting what the others will do; predictability is essential to team coordination. A fifth use of ShadowBox is to support leadership. For a given scenario, the leader or supervisor acts in the place of the panel of experts, and gets to see how the subordinates would act, and what their reasoning was; the idea is to strengthen the calibration of the leader and the subordinates. The subordinates don't have to agree with the supervisor, but they do have to understand what the supervisor expects and how the supervisor interprets the situation.

## Conclusion

The NDM framework has developed over the past 30 years, shaping the thinking and capabilities of a community of researchers and practitioners. The NDM community is now engaged in studying macrocognitive phenomena and developing methods for supporting these functions and processes. This work has shaped our thinking about cognitive processes such as decision making, sensemaking, and problem detection that are engaged in complex and uncertain environments. It has shaped our capabilities for training, decision support systems, and system design. NDM researchers typically work with domain specialists performing complex and challenging tasks. Accordingly, the methods and the models are especially suited to applications and are grounded in the variables that matter the most in “natural” conditions.

## Author contributions

GK is the lead author and took primary responsibility in preparing this manuscript. CW is the second author and helped with the writing and preparation.

### Conflict of interest statement

The authors declare that the research was conducted in the absence of any commercial or financial relationships that could be construed as a potential conflict of interest.
